# Hematohidrosis in Pediatric Practice – a Case Report and Review of the Literature

**DOI:** 10.15388/Amed.2024.31.1.2

**Published:** 2024-02-27

**Authors:** Oksana Matsyura, Lesya Besh, Svitlana Jefimova, Slivinska-Kurchak Khrystyna, Sergiy Gerasymov

**Affiliations:** 1 Danylo Halytsky Lviv National Medical University, Lviv, Ukraine Communal Nonprofit Enterprise “City Children’s Clinical Hospital of Lviv”, Lviv, Ukraine; 2 Communal Nonprofit Enterprise “City Children’s Clinical Hospital of Lviv”, Lviv, Ukraine

**Keywords:** hematohidrosis, case, child, diagnosis, treatment, hematohidrozė, atvejis, vaikas, diagnostika, gydymas

## Abstract

Hematohidrosis (bloody sweat) is a symptom of trophic damage to the vascular wall, in which sweat mixes with blood and seeps onto undamaged areas of the skin in the form of red or pink liquid (depending on the ratio of sweat to blood).

In our study we have analyzed 25 case reports of hematohidrosis in children, reported throughout the world using PubMed, ResearchGate with detailed description and opened access. We took into consideration: age of the patient, sex, location of bloody excretion, cause or trigger, treatment and its effectiveness.

Our clinical case present a 9-year-old girl complained of a periodic bleeding from the intact skin of the face, neck, thighs (without visible damage to the skin) manifested by red or pink liquid, nosebleeds, and bloody discharge from the mucous membrane of the eyes. The secretions were of varying intensity and lasted up to several hours. Most of all episodes are associated with a strong emotional exertion. One of the theories of hematohidrosis pathogenesis is evident vasoconstriction of the blood vessels surrounding the sweat glands, provoked by hyperactivation of the sympathetic nervous system, which is followed by their excessive vasodilation up to rupture and blood entering the sweat gland ducts. Capillary endothelial cells are known to contain β2-adrenoceptors, which, through the modulation of nitric oxide release, cause endothelium-dependent vasodilation. Blocking β-adrenoceptors (for example, propranolol) prevents excessive vasodilation of blood vessels and, accordingly, their rupture and blood flow to the sweat gland.

We managed to find out that the patient’s bloody sweat was a manifestation of a separate pathological phenomenon, and not one of the symptoms of another disease. A properly formed treatment complex and the great trust of the parents enabled to stabilize the child’s condition, and later to cure her. Currently, hematohidrosis is recognized as an independent disease that requires in-depth study of the triggering mechanisms of development, pathogenetic and clinical features.

## Abstract (it)

Hematidrozė (prakaitas su krauju) yra trofinio ląstelės sienelės sužalojimo simptomas, kai prakaitas susimaišo su krauju ir prasisunkia į nesužalotas odos vietas raudono ar rausvo skysčio pavidalu (spalva priklauso nuo prakaito ir kraujo santykio).

Šiame tyrime buvo išnagrinėtos 25 visame pasaulyje atliktos atvejų analizės, kai hematidrozė pasireiškė vaikystėje. Buvo panaudotos *PubMed* ir *ResearchGate* duomenų bazės, remtasi detaliais aprašais ir atvira prieiga. Atsižvelgėme į šiuos aspektus: paciento amžius, lytis, kraujingų išskyrų vieta, priežastis ar sukėlėjas, gydymas ir jo efektyvumas.

Mūsų klinikinis atvejis pristato 9 metų mergaitę, kuri skundėsi nuolatos besikartojančiu kraujavimu iš nesužalotos odos veido, kaklo ir šlaunų srityje (ant odos nebuvo jokių matomų sužalojimo požymių). Veržėsi raudonas arba rausvas skystis, bėgo kraujas iš nosies. Be to, iš gleivinės akių membranos veržėsi kraujingos išskyros. Šių išskyrų intensyvumas buvo įvairus, ir epizodai trukdavo po keletą valandų. Beveik po visų protrūkių buvo stiprus emocinis išsekimas. Viena iš hematidrozės patogenezės teorijų yra aiškiai matoma kraujagyslių, supančių prakaito liaukas, vazokonstrikcija, kurią sukelia simpatinės nervų sistemos hiperaktyvavimas. Tada būna perteklinė vazodilatacija. Kraujagyslės sutrūkinėja, ir kraujas patenka į prakaito liaukų latakus. Žinoma, kad kapiliarinės endotelinės ląstelės turi β2 adrenoreceptorius, kurie, moduliuodami azoto oksido išskyrimą, sukelia nuo endotelio priklausomą vazodilataciją. β adrenoreceptorių blokavimas (pavyzdžiui, panaudojant propranololį) neleidžia įvykti perteklinei kraujagyslių vazodilatacijai, ir todėl jos nesutrūkinėja. Tad kraujo tėkmė į prakaito liaukas yra nutraukiama.

Mums pavyko nustatyti, kad pacientės kraujingas prakaitas buvo atskiro patologinio reiškinio apraiška, o ne vienas iš kitos ligos simptomų. Tinkamai sudarytas gydymo kompleksas bei parodytas didžiulis pacientės tėvų pasitikėjimas leido stabilizuoti mergaitės būklę ir galiausiai ją pagydyti. Šiuo metu hematidrozė yra laikoma nepriklausoma liga, kuriai reikia išsamių tyrimų, norint išsiaiškinti, kokie mechanizmai ją sukelia ir skatina, bei kokios yra šios ligos patogeninės ir klinikinės savybės.

## Introduction

Hematohidrosis or hematidrosis (bloody sweat) is a rare clinical phenomenon, characterized by recurrent, spontaneous, self-limited episodes of bloody secretion from intact skin or mucosa in almost any part of the body, that are witnessed by medical personnel [1, 2]. In 83% of cases, it occurs in individuals 18 years old or younger [3]. The origin of this condition remains unknown but various causative factors have been suggested by Holoubek, like: systemic disease, vicarious menstruation, excessive exertion, psychogenic purpura. Acute fear and intense mental contemplation are the most frequent triggers of hematohidrosis [4, 5].

## Clinical case

A 9-year-old girl complained of a periodic bleeding from the intact skin of the face, neck, thighs (without visible damage to the skin) manifested by red or pink liquid, nosebleeds, and bloody discharge from the mucous membrane of the eyes. The secretions were of varying intensity and lasted up to several hours. All the listed symptoms were related to the psycho-emotional state of the child and mental exertion. These manifestations had been observed for several weeks before admission to hospital, they debuted after a family conflict.

The child consulted a family doctor, and was prescribed antihistamines, ascorutin in an outpatient setting. There was no improvement, episodes of bleeding became more frequent and the child was hospitalized for clarification of the diagnosis and further treatment in hospital.

**Examination on admission to hospital**. On examination: erythema on the face, occupying the region of the right cheek and forehead, bloody secretions on the intact skin of the face. Tonsils are pink, not enlarged, without plaques. Submandibular lymph nodes are palpable, mobile, not painful. On percussion over the lungs, there is a clear lung sound. During auscultation – vesicular breathing, rales are absent. Heart tones are rhythmic. The abdomen is soft, not painful, accessible for palpation in all regions. The lower margin of the liver is at the level of the costal arch. The spleen is not palpable. Pasternacki’s symptom is negative on both sides. Meningeal symptoms are negative.

Respiratory rate – 24/min, heart rate – 56/min, blood pressure – 100/60 mmHg.

### The child underwent the following clinical, laboratory and instrumental examinations

**A complete blood cell (CBC) count** was performed (Table 1), which showed borderline leukocytosis on the start of treatment and during active bleeding period versus normal values in 5 days dynamic. Leukocytosis can be associated with post-infectional changes and stress factor.

**Table 1:** A complete blood cell count results

Main parts of CBC	Start of treatment	5 days dynamic
Hemoglobin (g/dL)	12.2	12.9
Red blood cell count (million/cm^3^)	3.7	3.9
Platelet count (thousand/mm^3^)	268	200
White blood cell count (thousand/mm^3^)	12.3	6.3
	Differential (%)	
Neutrophils	62	54
Eosinophils	0	4
Lymphocytes	35	31
Monocytes	3	11

**A biochemical blood analysis** was performed (Table 2), which showed an elevated titer of antistreptolysin O antibody and means that patient had a recent strep infection.

**Table 2:** A biochemical blood analysis results

Test, units	Results	Normal ranges
Total protein, g/L	60.5	60–83
Total urea, mmol/L	5.79	2.1–8.5
Creatinine, mmol/L	74.7	61.9 to 114.9
Bilirubin, mmol/L	12.33	1.71–20.5
ALT, U/L	13.4	4–36
AST, U/L	19.1	8–33
Glucose (fasting), mmol/L	3.75	3.3–6.6
C-reactive protein, mg/dL	6	0.3–10
Rheumatoid factor	negative	negative
Antistreptolysin O titer, IU/ml	800	below 200

**Urinalysis**: pale-yellow color of urine (normal), specific gravity 1017 (normal range 1008–1030), protein negative (normal), blood negative (normal), 1–3 epithelial cells per visual field (normal range of renal tubular cells 0 to 3 per high power field), 1–2 white blood cells (normal range of renal tubular cells 0 to 4 per high power field).

**Coagulogram:** prothrombin time – 25 (normal range 12–14.8) seconds, prothrombin time ratio – 1.1 (normal range 0.9–1.1), recalcification time – 39 sec (normal range 27–41) sec, fibrinogen (Clauss) 4.4 (1.9–8.0) g/l.

**Microscopy of skin secretions on the face:** erythrocytes and salt crystals are present (normally absent).

**Faecal worm egg count:** no worm eggs and protozoan cysts detected (normal).

Ultrasound protocol of abdominal organs: within age-related normal ranges.

**ECG**: sinus rhythm, bradycardia – 56 beats per minute. During an orthostatic test heart rate increased to 80 beats per minute.

**Echocardiography**: The dimensions of the heart chambers are normal. The structure and function of the valves are within normal ranges. Septum defects are not detected. The flow of blood vessels is correct (normal).

### The child was consulted by specialists:

**ENT consultation** (bloody discharge periodically occurs in the ear area): within normal ranges.

**Ophthalmologist’s consultation** (blood discharge periodically occurs in the eye area): within normal ranges.

**Neurologist’s and psychiatrist’s consultation**: emotional lability. Psychocorrection is recommended.

**Genetic consultation**: no data about hereditary and metabolic disorders.

**Figure 1:**
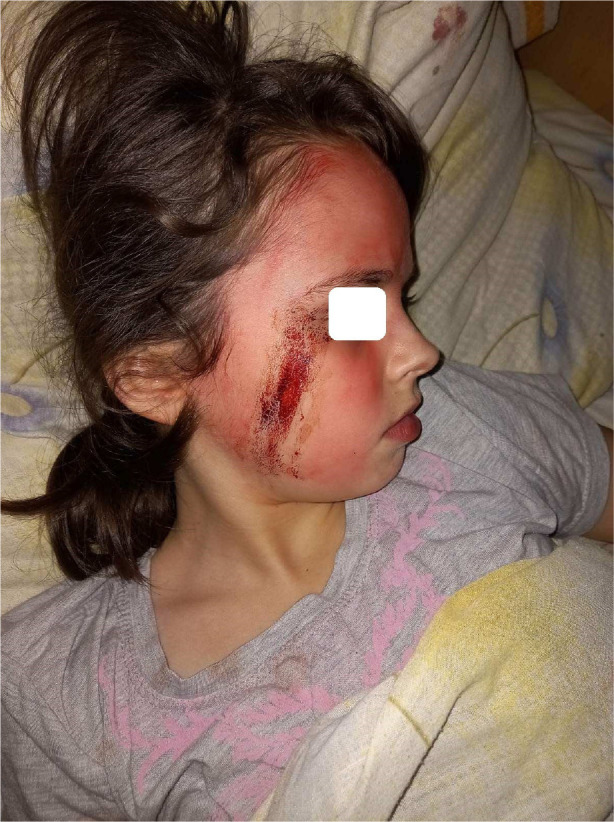
Sweat admixed with blood from the right side of the face.

**Figure 2:**
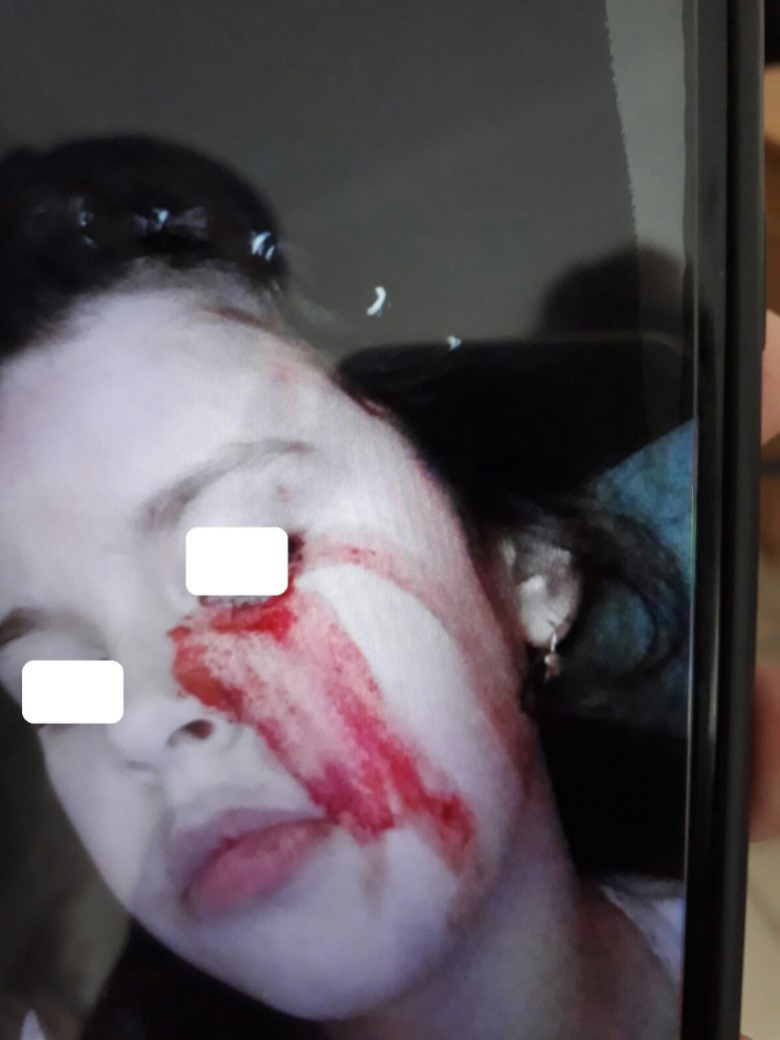
Sweat admixed with blood from the left eye.

**Figure 3:**
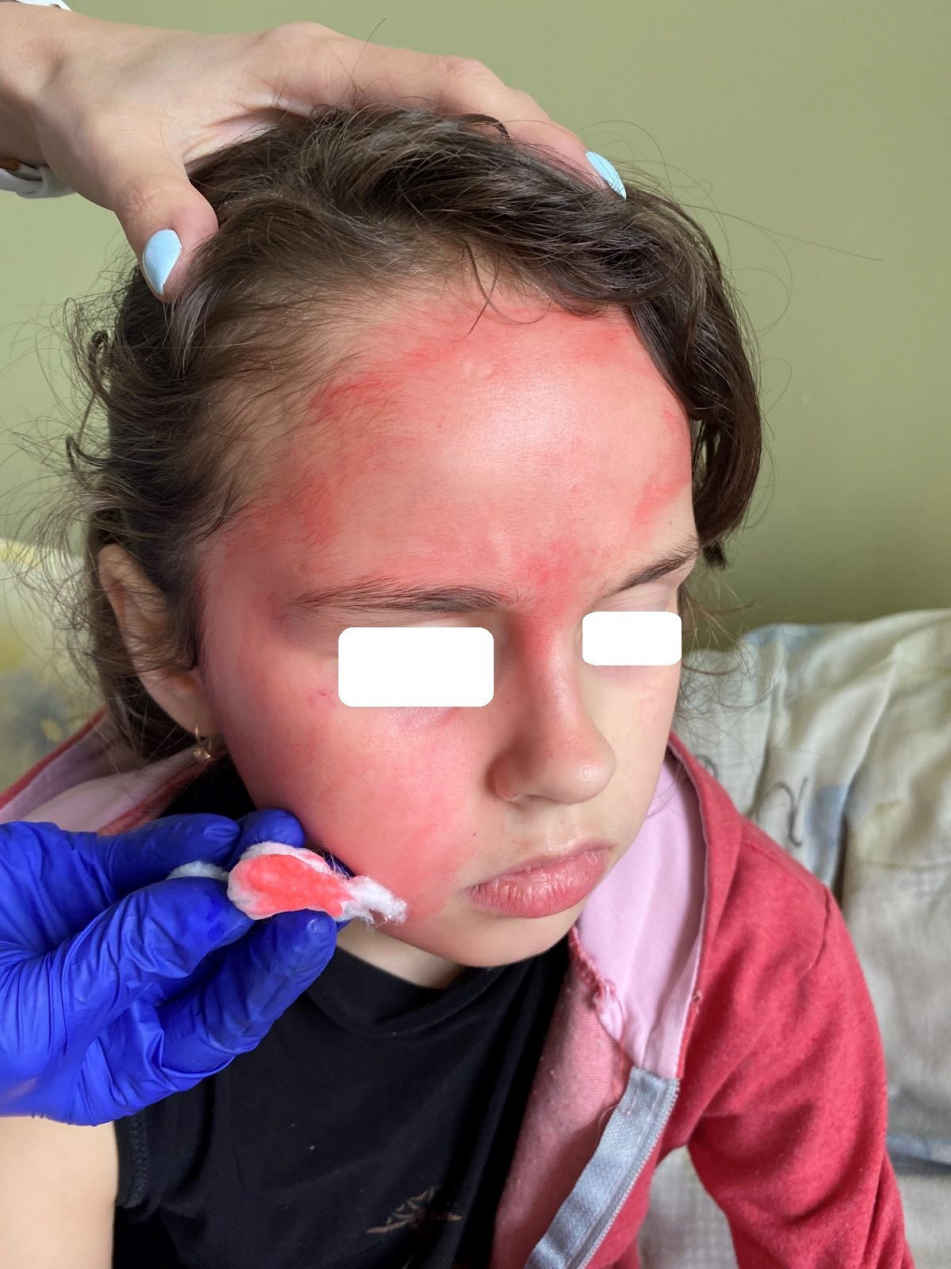
Hematohidrosis of the face.

**Figure 4:**
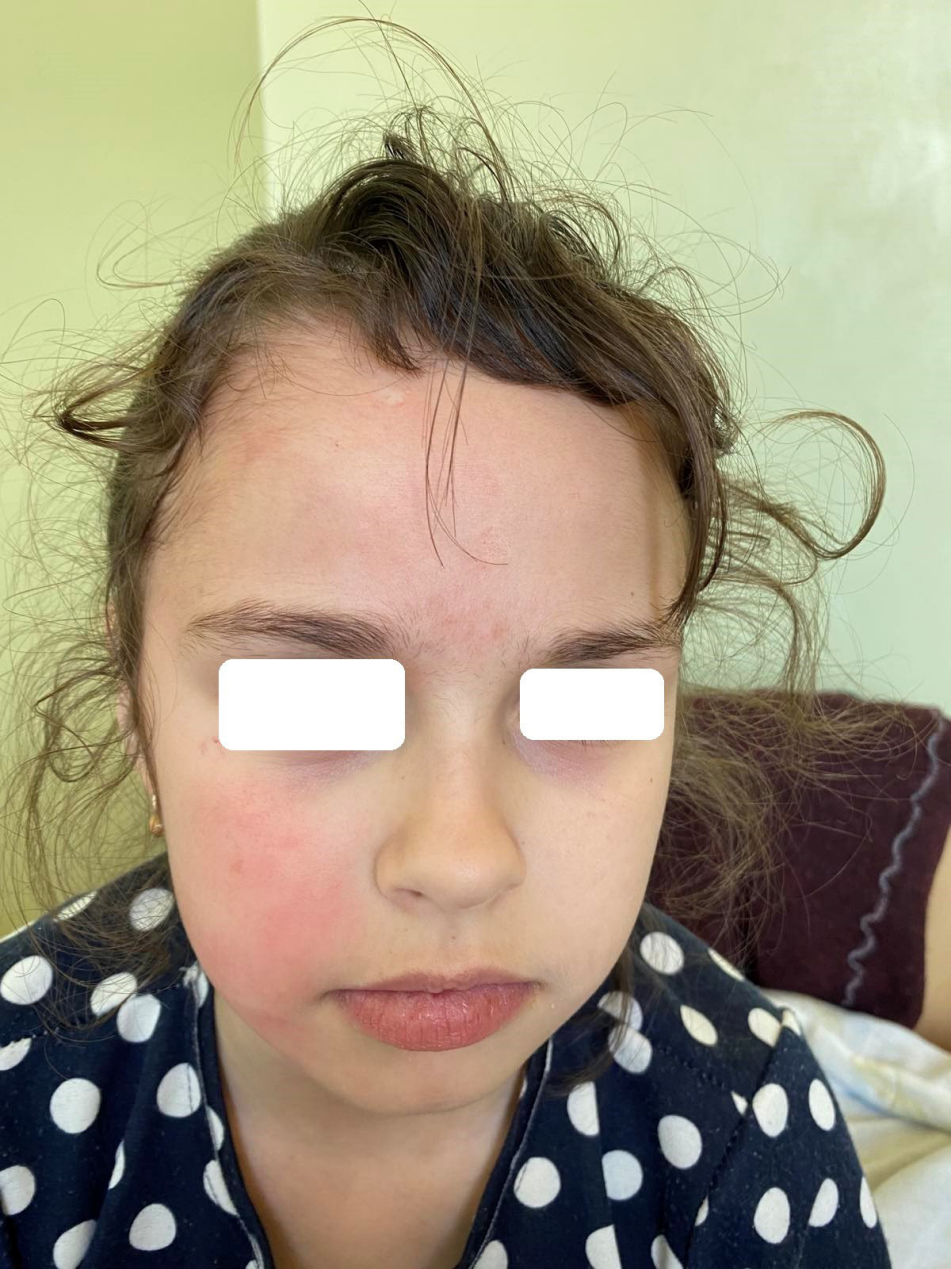
Face without skin damaging after treatment.

Figures 1–4 demonstrate changes on the skin.

Patient and parents signed an informed consent for publishing a case report and figures.

After conducting examinations, treatment was prescribed.

1. Anaprilin 10 mg twice a day along with control of blood pressure and ECG every 2 weeks.
2. Psychological correction – individual and family sessions with a psychologist.
3. Creation of a comfortable psycho-emotional environment in the family and educational establishment.
4. Treatment of the skin and mucous membranes with aseptic solutions during bleeding.

During the first month of treatment, the child’s condition improved, symptoms became rarer. At the 6th week of treatment, the dose of anaprilin was reduced and completely canceled in 3 months. Meetings with a psychologist were daily at the beginning of treatment, then twice a week, and further as needed.

Nowadays, a patient is feeling well. Manifestations of the disease regressed completely. The girl has learned to control her emotions, directs her energy into creativity and is a cheerful child.

Our team of doctors went through a «brainstorm» on the way to making a diagnosis. We considered many theories, from genetic abnormalities to symptom simulation. We managed to find out that the patient’s bloody sweat was a manifestation of a separate pathological phenomenon, and not one of the symptoms of another disease. A properly formed treatment complex and the great trust of the parents enabled to stabilize the child’s condition, and later to cure her. Currently, hematohidrosis is recognized as an independent disease that requires in-depth study of the triggering mechanisms of development, pathogenetic and clinical features.

## Review of the literature

Hematohidrosis is an extremely rare condition and the etiology of this disease is still unclear.

There are few theories, that explain the pathogenesis of hematohidrosis. The general consensus relates to intensified sympathetic activation due to extreme physical or emotional stress. It is found that the sympathetic fight-or-flight response to intense stress leads to constriction of capillary vessels feeding the sweat glands. When the anxiety subsides, the blood vessels dilate to the point of rupture, leading to the passage of blood through the ducts of the nearby sweat glands and presenting as droplets of blood mixed with sweat on the intact skin surface or mucosa in almost any part of the body [6, 7, 8].

Skin histopathological study by Zhang *et al*. revealed some intradermal bleeding and obstructed capillaries. No abnormality was found in sweat glands, hair follicles, and sebaceous glands. They concluded that pathological basis for hematohidrosis might be distinctive vasculitis [7].

The suggested diagnostic criteria for hematohidrosis are: (1) recurrent, spontaneous, painless and self-limited bloody secretion from skin or mucosa, witnessed and confirmed by health professionals, (2) the usual blood components are found on biochemistry studies of the discharge, and (3) the site of bleeding is intact with no abrasion, telangiectasia or purpura and after wiping the area, there is no evidence of oozing. All of these criteria must be met in order to rule out organic bleeding disorders, self-inflicted bleeding, factitious disorder, and chromhidrosis (colored sweat) [1].

The treatment of hematohidrosis still remains a challenge. There are some reports of successful use of propranolol. It is known, that capillaries have β2-adrenoreceptors on their endothelial cells, which through excretion of NO lead to endothelium-dependent vasodilatation. Block of β2-adrenoreceptors (β-blocker – propranolol) prevents excessive vasodilatation of blood vessels and, accordingly, their rupture and the flow of blood into the sweat gland. Propranolol is a nonselective blocker of β-adrenoceptors with membrane-stabilizing properties. The use of propranolol for the treatment of children with diseases of the cardiovascular system without age restrictions is regulated by Ministry of Health of Ukraine [9].

Several studies show transdermal atropine to be effective in diminishing the severity and frequency of bloody discharge episodes. Some authors recommend the administration of the antiepileptic drug, oxcarbazepine for resolving of symptoms [1].

In our study we have analyzed 25 case reports of hematohidrosis in children, reported throughout the world using PubMed, ResearchGate with detailed description and opened access. We took into consideration: age of the patient, sex, location of bloody excretion, cause or trigger, treatment and its effectiveness. All data was summarized in Table 2.

**Table 3:** Case reports concerning hematohidrosis in children

Case report	Age	Sex	Location of bloody excretion	Cause or trigger	Treatment	Effectiveness of treatment
The Turkish Journal of Pediatrics 2018 (1)	9 years	Girl	Forehead, eyes, ears, nails, arm, umbilical area, back, vagina, and gastrointestinal tract for several months	Extreme physical or emotional stress	Propranolol	Diminished frequency of episodes
The Turkish Journal of Pediatrics 2018 (1)	11 years	Girl	Ears lobules, nose, eyes	Unknown	Propranolol	Significant improvement in severity and frequency of the episodes
The Turkish Journal of Pediatrics 2018 (1)	8 years	Boy	Nail beds of his hands and feet	Upcoming exam, playing a computer game, watching fiction TV movies or when parents not satisfying his demands	No	Complete recover in 2 years
Case Reports in Dermatological Medicine, 2016 (10)	12 years	Girl	left side of the face, left eye, and tear duct	No	No	-
Indian J Dermatol 2013 (11)	12 years	Girl	forehead, scalp, cheek, nose, and trunk	No	Atropine transdermal patch	Gradual improvement in both the severity and frequency of the episodes
International Journal of Dermatology, 2008 (12)	13 years	Girl	Around the mouth	Strenuous exercise or prolonged exposure to heat	No	Spontaneous resolution over time
The Indian Journal of Pediatrics, 2012 (13)	10 years	Girl	Forehead, neck, umbilicus, wrists and legs, epistaxis	Stressful event	3 years of oral lorazepam with adverse side effects Propranolol	Improvement of symptoms
The American Journal of Otolaryngology- Head and Neck Medicine and Surgery, 2014 (14)	18 years	Girl	Bloody otorrhea	Stressful school life	No	Spontaneous resolution
Indian Journal of Psychiatry, 2014 (15)	10 years	boy	Umbilical, eyes, ear lobules, nose	Preexisting deviant disorder	Lorazepam, propranolol, behavioral therapy	Improvement of symptoms
Indian J Psychol Med. 2017 (16)	10 years	Girl	Skin of scalp	Underlying intense fear secondary to psychosocial stressor	Antidepressants, anxiolytics, psychotherapy	Remission
Bangladesh Journal of Child Health, 2021 (17)	12 years	Girl	Eyes, ear lobules, nose & navel	Stress	Propranolol	Improvement of symptoms
Indian Journal of Psychiatry, 2022 (19)	18 years	Girl	Eyes, ear, nose	During sleep or eating	Clonazepam + propranolol	Frequency of episodes diminished but persisted
					Olanzapine	symptoms resolved
Asian Archives of Pathology 2016 (20)	13 years	Girl	back, hands, legs and feet	No	Propranolol	Symptoms resolved
Indian Pediatrics, 2013 (21)	12 years	Girl	Face, limb, palm and sole but not from mucous membrane	No	Diazepam Propranolol	No significant improvement Symptoms resolved
Indian Journal of Dermatology, Venerology and leprology, 2009 (22)	12 years	Girl	Forehead, umbilical area	No	Tricyclic antidepressants	Mild improvement
Arch NIMH, 2020 (23)	13 years	Girl	Right nostril, right ear, right eye	Nightmares	Psychotherapy, propranolol	Improvement of symptoms
Indian Dermatol Online J. 2010 (24)	13 years	Boy	Forehead, lips, arms, trunk, external ear	Emotional conflicts	Relaxation, meditation	Improvement, but relapse immediately after stopping
Am J Clin Dermatol, 2010 (25)	13 years	Girl	Forehead, palms, nails, feet, thighs, trunk, tongue	No	Propranolol	Symptoms resolved
Arch Dermatol. 2012 (26)	18 years	Girl	Forehead, ears, nails, umbilicus	Stress	Propranolol	Improvement
Arch Pediatr. 2013 (27)	9 years	Girl	Forehead, chin, earlobe, pelvis	No	No	Spontaneous improvement
Indian Pediatr. 2013 (28)	12 years	Girl	Face, limb, palm, sole	No	Benzodiazepines Propranolol	No effect Symptoms resolved
Indian J Psychiatry. 2015 (29)	10 years	Girl	Eyes	Depression	Antidepressant Benzodiazepines Propranolol	Improvement
Int J Dermatol. 2015 (30)	18years	Girl	Forehead, palms, nails	Conversion, dissociative disorder	Propranolol	Improvement, sporadic recurrence
Int J Dermatol. 2016 (31)	9 years	Girl	Umbilicus, trunk, limbs	Child abuse	No	Healing in 1 year
Presse Med. 2016 (32)	11 years	Girl	Forehead, sculp, ears, umbilicus, trunk, vulva	Conversion	Anxiolytics Psychotherapy	Improvement

Most reported cases concerned children from Asia including India – 14 (56%) and Turkey – 4 (16%) with the rest from Bangladesh, South Africa, China, Thailand, Brazil, Tunisia. Only one case from Europe – Spain. Boys made up 12%, girls – 88%. The location of bloody excretion differed, but is mostly on face – forehead, eyes, ears, nails, trunk, umbilicus. Triggers were identified in 16 cases (64%), most of these – stress, among others: extreme physical excretion, prolonged exposure to heat, fear and nightmares. Three children had preexisting psychiatric disease: deviant disorder, dissociative disorder and depression. All children underwent detailed blood test and instrumental investigation to exclude congenital and acquired hemostatic disorder and vasculitis. The medical treatment included psychotherapy, antidepressants, anxiolytics, beta-blockers. Propranolol was effective in 13 cases (52%) and lead to significant improvement in severity and frequency of the episodes and further resolvent. But still 5 children recovered without treatment.

Hematohidrosis is a specific condition without definite and absolute criteria, specific laboratory or histological findings. But it should be taken into consideration when making a diagnosis in the case of bloody excretion from intact skin and mucosa with normal laboratory and instrumental examinations.
